# MAIR‐II deficiency ameliorates cardiac remodelling post‐myocardial infarction by suppressing TLR9‐mediated macrophage activation

**DOI:** 10.1111/jcmm.16070

**Published:** 2020-11-02

**Authors:** Saori Yonebayashi, Kazuko Tajiri, Nobuyuki Murakoshi, Dongzhu Xu, Siqi Li, Duo Feng, Yuta Okabe, Zixun Yuan, Zonghu Song, Kazuhiro Aonuma, Akira Shibuya, Kazutaka Aonuma, Masaki Ieda

**Affiliations:** ^1^ Graduate School of Comprehensive Human Sciences University of Tsukuba Tsukuba Japan; ^2^ Department of Cardiology Faculty of Medicine University of Tsukuba Tsukuba Japan; ^3^ Department of Immunology Faculty of Medicine University of Tsukuba Tsukuba Japan; ^4^ R&D Center for Innovative Drug Discovery University of Tsukuba Tsukuba Japan; ^5^ Life Science Center for Survival Dynamics Tsukuba Advanced Research Alliance (TARA) University of Tsukuba Tsukuba Japan

**Keywords:** inflammation, myocardial infarction, remodelling

## Abstract

Macrophages are fundamental components of inflammation in post‐myocardial infarction (MI) and contribute to adverse cardiac remodelling and heart failure. However, the regulatory mechanisms in macrophage activation have not been fully elucidated. Previous studies showed that myeloid‐associated immunoglobulin–like receptor II (MAIR‐II) is involved in inflammatory responses in macrophages. However, its role in MI is unknown. Thus, this study aimed to determine a novel role and mechanism of MAIR‐II in MI. We first identified that MAIR‐II–positive myeloid cells were abundant from post‐MI days 3 to 5 in infarcted hearts of C57BL/6J (WT) mice induced by permanent left coronary artery ligation. Compared to WT, MAIR‐II–deficient (*Cd300c2* ^−/−^) mice had longer survival, ameliorated cardiac remodelling, improved cardiac function and smaller infarct sizes. Moreover, we detected lower pro‐inflammatory cytokine and fibrotic gene expressions in *Cd300c2* ^−/−^‐infarcted hearts. These mice also had less infiltrating pro‐inflammatory macrophages following MI. To elucidate a novel molecular mechanism of MAIR‐II, we considered macrophage activation by Toll‐like receptor (TLR) 9–mediated inflammation. In vitro, we observed that *Cd300c2* ^−/−^ bone marrow–derived macrophages stimulated by a TLR9 agonist expressed less pro‐inflammatory cytokines compared to WT. In conclusion, MAIR‐II may enhance inflammation via TLR9‐mediated macrophage activation in MI, leading to adverse cardiac remodelling and poor prognosis.

## 1 INTRODUCTION

Myocardial infarction (MI) is a life‐threatening event as a result of ischaemic cardiovascular disease, which remains to be one of the highest causes of mortality despite available drug therapy and treatment.^1^ Following MI, monocytes and macrophages can co‐ordinate inflammation, healing and left ventricular (LV) remodelling.^2^, ^3^, ^4^ Dysregulated macrophage activation has been shown to cause adverse cardiac events including enlarged infarct size and exacerbated fibrosis.^5^, ^6^ Also, depletion and injection studies have shown the presence of macrophages is necessary for proper healing to occur.^7^, ^8^ Therefore, the careful orchestration of their inflammatory and healing properties is essential for optimal healing. However, the underlying mechanisms in macrophage activation to regulate the post‐MI response have not yet been fully elucidated.

Myeloid‐associated immunoglobulin‐like receptor II (MAIR‐II) (also known as CD300c2, DIgR1, LMIR‐2 and CLM‐4) is encoded by *AF2051705* and a part of the CD300 multigene family.^9^ The CD300 family in humans consists of 8 genes on chromosome 17,^10^ whereas in mice there are 9 genes on chromosome 11.^9^ MAIR‐II is an activating cell surface receptor expressed on macrophages and a subset of B cells in the spleen and peritoneal cavity.^11^, ^12^ MAIR‐II associates with the signalling adaptor DAP12 and FcRγ chain, which carry the immunoreceptor tyrosine–based activating motif in peritoneal macrophages.^13^ Through these interactions, MAIR‐II mediates the activation of pro‐inflammatory cytokine production of IL‐6 and TNF‐α in macrophages.^11^, ^12^ As such, the role of MAIR‐II in inflammation has been demonstrated in some disease models. Our group previously reported that in a mouse sepsis model, MAIR‐II deficiency led to decreased inflammatory monocyte migration to sites of infection.^13^ Similarly in a pulmonary fibrosis mouse model, we showed that MAIR‐II deficiency resulted in a lower inflammatory response in alveolar macrophages.^14^ However, the role of MAIR‐II in inflammatory responses in MI remains unknown.

In our study, we investigated the role of MAIR‐II in MI. We found that MAIR‐II deficiency leads to favourable cardiac outcome including longer survival, smaller infarct sizes and better cardiac function post‐MI. Moreover, MAIR‐II amplifies CpG oligodeoxynucleotide (CpG‐ODN)‐Toll‐like receptor (TLR) 9–mediated macrophage activation to produce pro‐inflammatory cytokines.

## 2 MATERIALS AND METHODS

### 2.1 Animals

C57BL/6J were purchased from CLEA Japan (Tokyo, Japan). MAIR‐II–deficient (*Cd300c2* ^−/−^) mice were originally backcrossed onto the C57BL/6J genetic background for 12 generations^15^ and then bred in our laboratory. All mice used for the experiments were male. All animal experiments were approved by the Institutional Animal Experiment Committee of the University of Tsukuba and conform to the NIH Guide for the Care and Use of Laboratory Animals.

### 2.2 MI Model

MI was induced on male mice approximately 10‐12 weeks old that weighed at least 25 g. The classical MI method was followed as previously described.^16^ Briefly, mice were anaesthetized by intraperitoneal injection with ketamine/xylazine (100‐120 mg/kg bodyweight for ketamine and 7‐8 mg/kg bodyweight for xylazine), intubated and connected to a ventilator (Mouse Ventilator Minivent Type 845; Harvard Apparatus). The chest cavity was opened via left thoracotomy to expose the heart, allow the left anterior descending coronary artery to be visualized by eye or magnifying glasses, and permanently ligated with a 7‐0 nylon suture at the site of its emergence from the left atrium. Complete occlusion of the vessel was confirmed by the presence of myocardial blanching in the perfusion bed. Mice that died during recovery from anaesthesia were excluded from the analysis. For sham mice, the same MI induction procedure was performed, but without a left anterior descending artery ligation.

### 2.3 Survival analysis

A total of 72 mice were used in the survival analysis (wild‐type [WT] sham, n = 5; *Cd300c2* ^−/−^ sham, n = 5; WT MI, n = 33; *Cd300c2* ^−/−^ MI n = 29). To reduce the suffering of mice, we set humane end‐points to decide when to kill the mice as previously described.^16^ The humane end‐points included body temperature and physical activity that were significantly worse than the active mice including a decrease or no increase in a few hours, the mice did not respond to 3 intermittent stimulations in half an hour, and the respiratory rate of mice were abnormally rapid or slow, or rapid or progressive weight loss (a 20% loss of bodyweight). Mice were killed by lethal intraperitoneal injection of sodium pentobarbital (200 mg/kg) or CO_2_ inhalation when they reached these end‐points to avoid further distress. Only the animals reaching the end‐point before the evaluation at the end of the study were considered valid and used in the survival analysis. Mice care was maintained by trained staff. Mice were monitored every 12 hours after surgery. After 14 days, all mice that survived were killed by lethal intraperitoneal injection of sodium pentobarbital (200 mg/kg) or CO_2_ inhalation.

### 2.4 Echocardiography

Transthoracic echocardiography was performed with a Vevo 2100 instrument (Fujifilm Visual Sonics) equipped with an MS‐400 imaging transducer. Isoflurane induction was performed in an induction box with 3% isoflurane in pure medical oxygen. After the mouse's righting reflex waned, it was fixed in the supine position on a heating pad to maintain normothermia, followed by electrocardiographic limb electrodes being placed. Anaesthesia was maintained at 1% isoflurane. Echocardiography parameters were measured including interventricular septum thickness in end diastole (IVSd), left ventricular posterior wall in diastole (LVPWd), left ventricular diameter at end diastole (LVDd), fractional shortening (FS) and ejection fraction (EF).

### 2.5 Histopathological examination

The hearts were fixed in 4% paraformaldehyde in phosphate‐buffered saline and embedded in paraffin wax. For infarct area evaluation, 3‐μm‐thick sections were cut and Masson's trichrome staining was performed. Infarct size was calculated by using the length‐based method^17^ with the following formula: (*x*/*y* + *a*/*b*)/2, where x and y represent the outer length of the fibrotic area and the total outer perimeter of the heart section, respectively, and a and b represent the inner length of the fibrotic area and the total inner perimeter of the heart section, respectively. The slides were observed with a virtual slide scanner (NanoZoomer, Hamamatsu Photonics). Analysis was performed by using NDR.view2 (Hamamatsu Photonics). Three microscopic fields were randomly chosen (BZ‐9000; Keyence), and the areas of fibrosis, capillaries, arterioles and cardiomyocytes in the infarct areas were measured and analysed using analysis software (Image J, version 1.53).

### 2.6 Flow cytometry

The excised hearts were minced with fine scissors into 1‐2 mm^3^ pieces. The minced heart tissue was transferred into a tube containing enzyme solution (200 units/mL collagenase II [Worthington], 500 units/mL hyaluronidase type IV‐S [Sigma] and 100 units/mL DNase I [Takara Biotec]) and dissociated using the gentleMACS™ Dissociator (Miltenyi Biotec). For flow‐cytometric analysis, the dissociated cells were stained directly by using fluorochrome‐conjugated mouse‐specific antibodies and analysed with a FACSVerse™ instrument (BD Biosciences) using FlowJo version 10.2 software (Tree Star). Cells that did not stain with 7‐AAD (Tonbo Biosciences) were deemed viable. The antibodies used were as follows: anti‐CD45 (clone 30‐F11; eBioscience), anti‐c‐Kit (clone 2B8; BioLegend), anti‐Sca‐1 (clone E13‐161.7; BioLegend), anti‐Ly6G (clone RB6‐8C5, TONBO Biosciences), anti‐CD11b (clone M1/70, eBioscience), anti‐F4/80 (clone BM8.1; Tonbo Biosciences), anti‐CD206 (clone C068C2, BioLegend) and anti‐MAIR‐II (clone TX52; BioLegend).

### 2.7 RNA extraction and quantitative reverse‐transcription polymerase chain reaction (qRT‐PCR)

All hearts removed for qRT‐PCR were snap‐frozen and stored at −80°C. For preparation of total RNA, the tissue was homogenized using a bead kit (MagNA Lyser Green Beads; Roche Diagnostics) according to the manufacturer's instructions. Total RNA samples from heart tissue and cultured cells were prepared by using an RNeasy Mini Kit (Qiagen). Complementary DNA was synthesized from 1 μg total RNA with a High‐Capacity cDNA Reverse Transcription Kit (Applied Biosystems). qRT‐PCR analysis was performed using the LightCycler® 480 System (Roche Applied Science) with a Universal Probe Library (Roche Applied Science). Hypoxanthine‐guanine phosphoribosyltransferase (*Hprt*) RNA was used as an internal control. Gene expression values were calculated by using the 2^−Δ^ *^C^* ^t^ method.

### 2.8 Enzyme‐Linked Immunosorbent Assay (ELISA)

The concentrations of cytokines and chemokines in the culture supernatants were measured with DuoSet ELISA Kits (R&D Systems).

### 2.9 Analysis of the level of free circulating blood mitochondrial DNA (mtDNA)

Blood samples were collected with a heparin‐coated syringe to obtain plasma from mice 1 day after MI or sham operation. DNA was extracted from cell‐free, platelet‐poor plasma using QIAamp MinElute ccfDNA Mini Kit (Qiagen). The quantitative analysis of mtDNA was conducted by using the qRT‐PCR method. PCR primers were designed to target gene sequences in murine mtDNA: NADH dehydrogenase subunit 6.

#### 2.9.1 Cell culture of bone marrow–derived macrophages

To induce bone marrow (BM)–derived macrophages (BMDMs), we first cultured BM cells from approximately 7‐ to 8‐week‐old WT and *Cd300c2* ^−/−^ mice with RPMI‐1640 medium supplemented with 10 ng/mL recombinant murine macrophage colony‐stimulating factor (R&D Systems), 10% heat‐inactivated foetal bovine serum, penicillin (100 U/mL) and streptomycin (100 μg/mL) in a humidified incubator at 37°C and 5% CO_2_. On day 5 of culture, BMDMs were stimulated with 100 nM of ODN1668 (InvivoGen) with or without 100 nM of ODN2088 (InvivoGen). The cells were harvested after 24 hours of stimulation.

#### 2.9.2 Statistical analysis

All data are expressed as mean ± standard error of the mean (SEM). Normality was verified with the Shapiro‐Wilk test. Statistical analyses were performed using an unpaired two‐tailed *t* test or Mann‐Whitney *U* test for experiments comparing two groups. For multiple comparisons, one‐way analysis of variance (ANOVA) with a Tukey post hoc test or a Kruskal‐Wallis analysis with a post hoc Steel‐Dwass or Steel test was used. Survival distributions were estimated by the Kaplan‐Meier method and compared by the log‐rank test. A *P* < .05 was considered statistically significant. All statistics were calculated with JMP software (SAS Institute).

## 3 RESULTS

### 3.1 MAIR‐II^+^ myeloid cells infiltrate into the heart post‐MI

**Figure 1:**
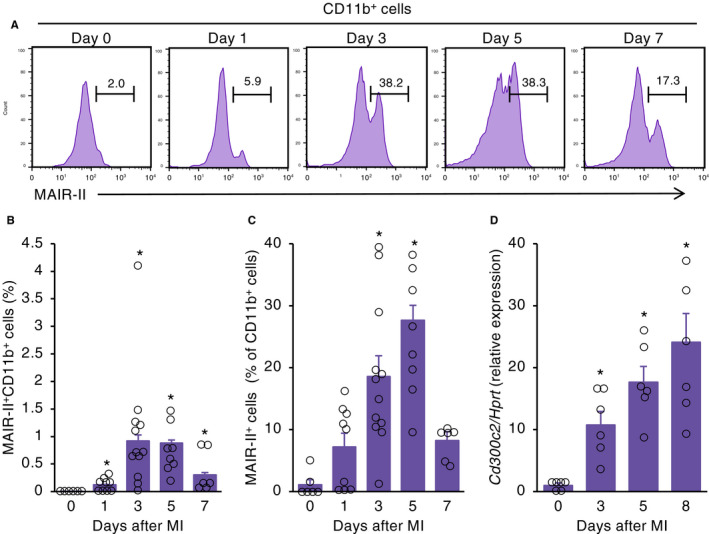
MAIR‐II^+^ myeloid cells infiltrate into infarcted myocardium. A, Flow‐cytometric analysis of infiltrating MAIR‐II^+^CD11b^+^ myeloid cells in WT hearts on days 0, 1, 3, 5 and 7 after MI. B, C, Quantification of flow cytometry of (B) MAIR‐II^+^CD11b^+^ cells and (C) MAIR‐II^+^ cells among CD11b^+^ cells in infarcted hearts on days 0, 1, 3, 5 and 7 after MI (n = 6‐12 at each time‐point). Results are presented as mean ± SEM. *P < .05 vs day 0 by Steel's method. D, mRNA expression of MAIR‐II (*Cd300c2*) in hearts obtained from MI mice at the indicated time‐points. Results are reported as the fold change in gene expression relative to baseline expression (n = 6‐7 at each time‐point). The values are expressed as mean ± SEM. *P < .05 by Kruskal‐Wallis analysis with a post hoc Steel test

We first examined the dynamics of inflammatory cells in post‐MI hearts. We found that CD45^+^ leucocytes, especially CD11b^+^ myeloid cells (both Ly6G^high^ and Ly6G^low^), significantly increased in the heart after MI (Figure S1A,B). Then, we examined MAIR‐II expression on myeloid cells in infarcted myocardium. We observed that MAIR‐II^+^CD11b^+^ cells peaked from days 3‐5 post‐MI (Figure 1A,B). There was also a gradual increase in MAIR‐II^+^ cells among CD11b^+^ cells until day 5 after MI (Figure 1C). Moreover, we confirmed that MAIR‐II had no expression in cardiac troponin T^+^ cardiomyocytes, vimentin^+^ fibroblasts and CD31^+^ endothelial cells (Figure S2). Furthermore, MAIR‐II mRNA expression increased progressively until day 8 following MI (Figure 1D).

### 3.2 MAIR‐II deficiency leads to favourable cardiac outcome after MI

**Figure 2:**
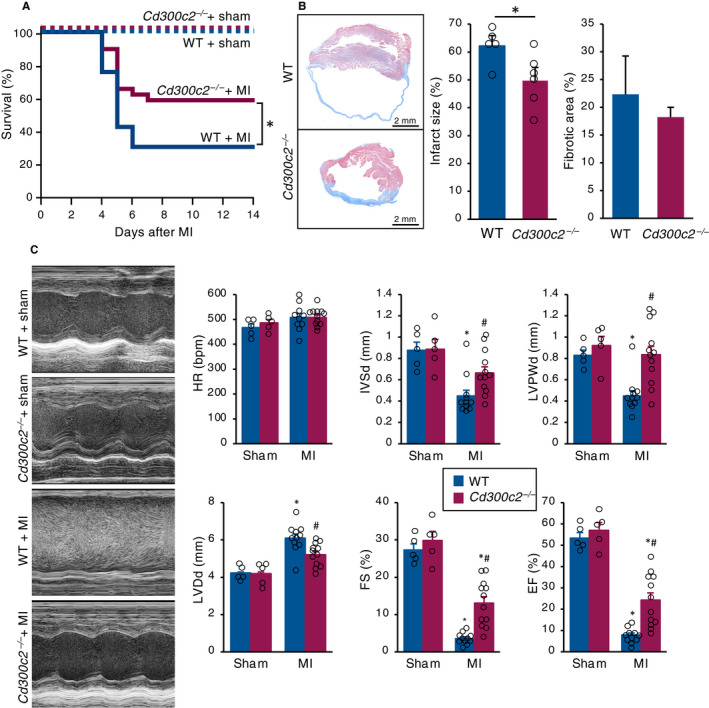
MAIR‐II deletion attenuates adverse remodelling after MI. A, The Kaplan‐Meier survival analysis 14 days after MI or sham operation. Blue line for WT with MI (n = 33), red line for *Cd300c2* ^−/−^ with MI (n = 29), dotted blue line for WT with sham operation (n = 5) and dotted red line for KO with sham operation (n = 5). *P < .05 by log‐rank. B, Representative cross sections of heart tissue one month after MI and the quantification of infarct sizes and fibrotic areas between WT and *Cd300c2* ^−/−^. Results are presented as mean ± SEM, n = 5‐6 each, *P < .05 by the Mann‐Whitney *U* test. C, Representative M mode images of echocardiography between sham and post‐MI day 8 in WT and *Cd300c2* ^−/−^ mice. Bar graphs represent echocardiographic parameters from the indicated animals. Results are presented as mean ± SEM, n = 5‐11, *P < .05 vs sham, #*P* < .05 vs WT by the Kruskal‐Wallis analysis with a post hoc Steel‐Dwass test

To investigate the role of MAIR‐II in MI, we compared WT and *Cd300c2* ^−/−^ mice in several cardiac parameters after MI induction. In post‐MI survival, *Cd300c2* ^−/−^ mice survived longer following MI compared to WT mice (Figure 2A). Moreover, *Cd300c2* ^−/−^‐infarcted hearts had smaller infarct sizes compared to WT hearts (Figure 2B and Figure S3A). However, the extent of fibrosis was similar between WT and *Cd300c2* ^−/−^ (Figure 2B and Figure S3A). After further investigation, we found there were more surviving cardiomyocytes in the infarct areas of *Cd300c2* ^−/−^ mice than in that of WT mice (Figure S3B,C). There were no differences in the amount of capillaries and arterioles between WT and *Cd300c2* ^−/−^(Figure S3C). In echocardiography, *Cd300c2* ^−/−^ had less severe cardiac remodelling in IVSd, LVPWd and LVDd (Figure 2C). Also, *Cd300c2* ^−/−^ had improved cardiac function in FS and EF compared to WT (Figure 2C).

### 3.3 MAIR‐II deficiency decreases pro‐inflammatory macrophage influx following MI

**Figure 3:**
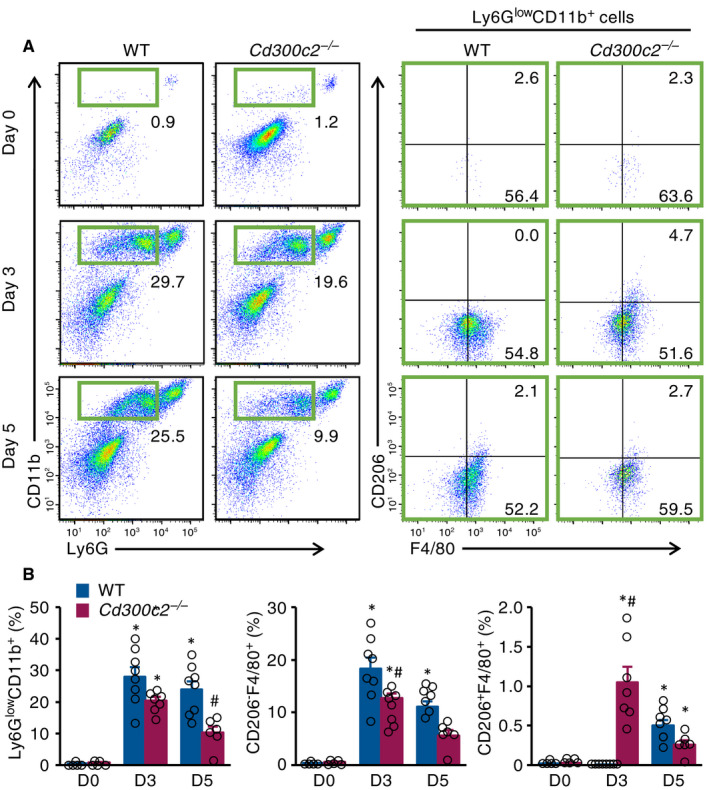
MAIR‐II deficiency reduces pro‐inflammatory macrophage influx. A, Representative flow‐cytometric plots showing Ly6G^low^CD11b^+^ macrophages, CD206^‐^F4/80^+^ pro‐inflammatory macrophages and CD206^+^F4/80^+^ anti‐inflammatory macrophages from WT and *Cd300c2* ^−/−^ hearts on days 0, 3 and 5 after MI. B, Quantification of Ly6G^low^CD11b^+^ macrophages, CD206^‐^F4/80^+^ pro‐inflammatory macrophage and CD206^+^F4/80^+^ anti‐inflammatory macrophages as a percentage of live cells from WT and *Cd300c2* ^−/−^ hearts on days 0, 3 and 5 after MI. Results are presented as mean ± SEM, *P < .05 vs day 0, #*P* < .05 vs WT by one‐way ANOVA with Tukey's post hoc test

To observe the effect of MAIR‐II deficiency on post‐MI macrophage infiltration, flow‐cytometric analyses were performed. In *Cd300c2* ^−/−^‐infarcted hearts, there were significantly fewer Ly6G^low^CD11b^+^ macrophages on post‐MI day 5 (Figure 3A,B). There was also a marked decrease in pro‐inflammatory CD206^‐^F4/80^+^ macrophage infiltration in *Cd300c2* ^−/−^ infarcted hearts on day 3 following MI (Figure 3A,B). Moreover, anti‐inflammatory CD206^+^F4/80^+^ macrophage infiltration significantly increased in *Cd300c2* ^−/−^ infarcted hearts on day 3 post‐MI (Figure 3A,B). The infiltration of Ly6G^high^CD11b^+^ neutrophils and Lin^‐^c‐kit^+^Sca‐1^+^ haematopoietic stem cells was also comparable between WT and *Cd300c2* ^−/−^ mice (Figure S4 and Figure S5).

### 3.4 MAIR‐II deficiency lowers inflammatory cytokine and fibrosis‐related gene expressions in infarcted hearts

**Figure 4:**
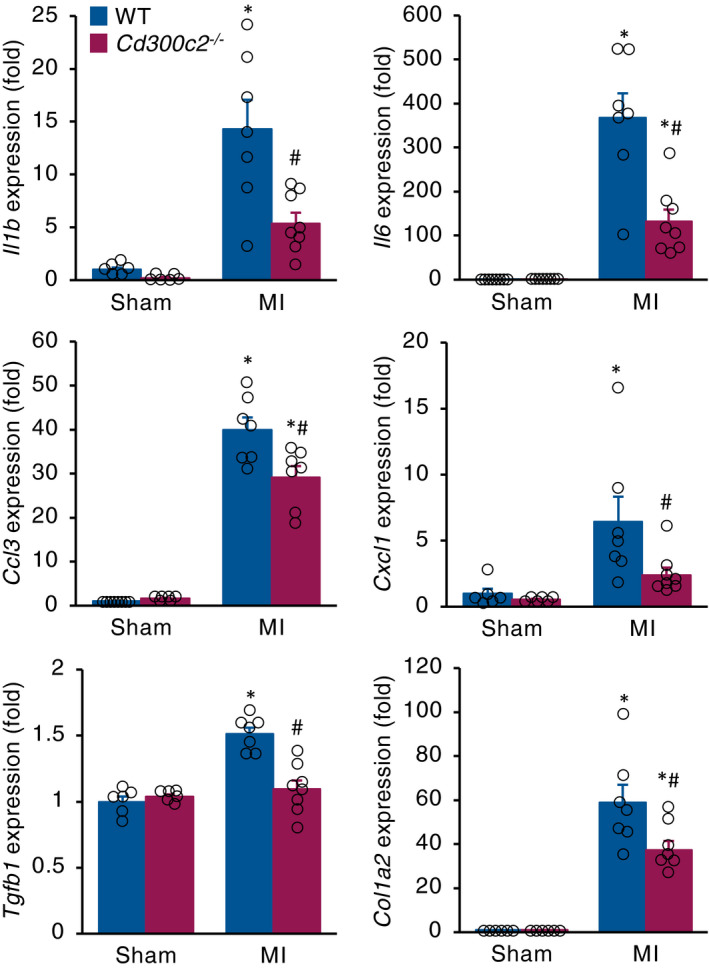
Inflammatory cytokine and fibrosis‐related gene expression in infarcted hearts. mRNA expression of inflammatory and fibrotic markers in hearts from WT and *Cd300c2* ^−/−^ hearts post‐MI day 5. Results are presented as mean ± SEM, n = 6‐8, *P < .05 vs day 0, #*P* < .05 vs WT by one‐way ANOVA with Tukey's post hoc test

We then investigated the cytokine and chemokine milieu in infarcted hearts. The heart homogenates from *Cd300c2* ^−/−^ mice had significantly reduced mRNA levels of the pro‐inflammatory cytokines IL‐1β and IL‐6 and chemokines CCL3 and CXCL1 (Figure 4). Also, *Cd300c2* ^−/−^ caused less gene expression of fibrotic markers TGF‐β and collagen type I alpha 2 (Figure 4). mRNA levels of MMP2 and MMP9 were comparable between WT and *Cd300c2* ^−/−^ mice (Figure S6).

### 3.5 MAIR‐II enhances CpG‐ODN‐TLR9–mediated macrophage activation

**Figure 5:**
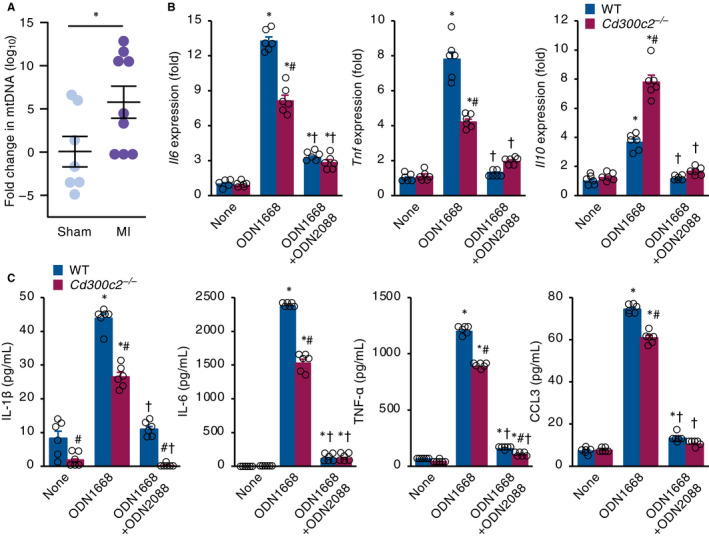
MAIR‐II up‐regulates CpG‐ODN‐TLR9–mediated macrophage activation. A, The plasma levels of circulating mtDNA were measured 1 day after MI or sham operation. Results are reported as the fold change in mtDNA amount relative to sham. The values are expressed as mean ± SEM, n = 7‐9, *P < .05 by an unpaired two‐tailed *t* test. B, C, Quantification of pro‐inflammatory and anti‐inflammatory cytokine expressions by (B) qRT‐PCR and (C) ELISA in WT and *Cd300c2* ^−/−^ BMDMs with none, ODN1668 and ODN1668 + ODN2088 stimulation. Results are presented as mean ± SEM, n = 6, * *P* < .05 vs none, # *P* < .05 vs WT, ^†^ *P* < .05 vs ODN1668 by one‐way ANOVA with Tukey's post hoc test

Upon MI, necrotic myocardial cells release several intracellular molecules called danger‐associated molecular patterns (DAMPs), such as mtDNA, into the extracellular space causing sterile inflammation.^18^ Elevated levels of circulating mtDNA in blood have been reported in MI patients and mice.^19^ Moreover, patients with acute coronary syndrome and increased circulating levels of mtDNA is a predictor of lethality.^20^ In our MI mouse model, we observed that plasma levels of circulating mtDNA significantly increased in MI mice compared to sham‐operated mice (Figure 5A).

Similar to bacterial DNA, mtDNA is enriched with unmethylated CpG motifs, which induce elevated inflammation through TLR9 activation.^21^ Because unmethylated CpG‐ODN binds to TLR9 on macrophages and strongly activates them to produce pro‐inflammatory cytokines,^22^ we investigated the effect of MAIR‐II on CpG‐ODN–mediated macrophage activation. In *Cd300c2* ^−/−^ BMDMs stimulated by a TLR9 agonist ODN1668, lower mRNA expressions of pro‐inflammatory cytokines IL‐6 and TNF‐α and higher expression of anti‐inflammatory cytokine IL‐10 were detected compared to WT BMDMs (Figure 5B). ELISA protein analysis also showed lower IL‐1β, IL‐6, TNF‐α and CCL3 in MAIR‐II KO BMDMs stimulated by ODN1668 compared to WT BMDMs (Figure 5C). When we inhibited TLR9‐mediated signalling by using a TLR9 antagonist ODN2088, BMDMs from WT and *Cd300c2* ^−/−^ mice produced comparable or relatively comparable levels of pro‐inflammatory and anti‐inflammatory cytokines (Figure 5B,C).

## 4 DISCUSSION

In this investigation, we have revealed a novel role and mechanism of MAIR‐II in MI. When MAIR‐II is deficient, post‐MI inflammation is attenuated and cardiac remodelling is alleviated. These were followed by longer survival times, smaller infarct sizes and improved cardiac function post‐MI. Furthermore, MAIR‐II increases pro‐inflammatory cytokine production via CpG‐ODN‐TLR9–mediated macrophage activation.

MAIR‐II is gaining attention as a novel regulator of inflammation through various molecular mechanisms in monocytes and macrophages. We previously found that MAIR‐II expressed on inflammatory monocytes regulates lipopolysaccharide‐TLR4–mediated cell adhesion to vascular cell adhesion molecule 1, promoting monocyte migration to sites of infection.^13^ We also previously showed that MAIR‐II amplified high mobility group box 1–mediated TLR4 inflammatory responses in alveolar macrophages to produce neutrophil chemoattractants, resulting in augmented neutrophil accumulation and inflammation.^14^ In our current study, we have revealed that MAIR‐II enhances CpG‐ODN‐TLR9–mediated pro‐inflammatory cytokine production in BMDMs. Our data suggest that diminishing MAIR‐II’s signalling through TLR9 in macrophages would be a promising target to reduce excess inflammation following MI.

Macrophages are one of the major players in orchestrating inflammatory and fibrotic responses following MI. Interfering with or extremely altered macrophage responses in either inflammation or fibrosis have led to detrimental consequences.^23^, ^24^ In our investigation, we demonstrated that MAIR‐II deficiency reduced pro‐inflammatory and pro‐fibrotic cytokine expressions in hearts on days 3 and 5 after MI, which was observed with more favourable LV remodelling and survival. A lower influx of pro‐inflammatory macrophages on days 3 and 5, and a greater influx of anti‐inflammatory macrophages on day 3 after MI were also detected. These results indicate that MAIR‐II induces an early pro‐inflammatory macrophage influx and a late anti‐inflammatory macrophage influx, thus exacerbating post‐MI inflammation.

We found that the length of infarct segments was significantly shorter in *Cd300c2* ^−/−^ mice than in WT mice. After further investigation, we found there were more surviving cardiomyocytes in the infarcted segments of *Cd300c2* ^−/−^ mice than in that of WT mice. From these findings, the infarcted area with fewer remaining cardiomyocytes may be vulnerable to wall stress and thus become more stretched. In this study, we could not clarify the exact mechanism by which MAIR‐II deficiency decreases myocardial necrosis. However, one possible mechanism is the suppression of post‐MI inflammation associated with macrophage inactivation by MAIR‐II deficiency. Previous studies have shown that post‐MI inflammation is essential for the healing process, but excessive inflammation is associated with cardiac rupture, ventricular aneurysm formation and exacerbation of LV remodelling.^25^, ^26^ Macrophage activation and polarization have also received considerable attention in relation to post‐infarction inflammation. In this study, we found that MAIR‐II enhanced TLR9‐mediated activation of macrophages that produced a significant amount of pro‐inflammatory cytokines. Thus, macrophage activation via MAIR‐II may have caused excessive post‐MI inflammation and exacerbated cardiomyocyte necrosis.

TLRs are pattern‐recognition receptors that recognize DAMPs, which are endogenous molecules with specific intracellular functions that are released from necrotic cells or secreted from damaged living cells.^27^ In MI, mtDNA is released upon cardiac cell death and acts as a DAMP, inducing TLR9‐dependent NF‐κB activation.^18^ Once NF‐κB is activated, pro‐inflammatory cytokine transcription is up‐regulated.^28^ Moreover, the role of TLR9 has been implicated in several animal studies of cardiovascular diseases including myocardial infarction,^29^, ^30^, ^31^ myocardial ischaemia/reperfusion injury,^32^ atherosclerosis^33^, ^34^ and heart failure.^35^, ^36^ Elevated plasma levels of mtDNA have also been reported in patient studies associated with increased mortality and cardiac inflammation.^20^, ^37^ In our study, the amount of circulating mtDNA increased following MI and lower pro‐inflammatory cytokine expression was observed in MAIR‐II–deficient BMDMs stimulated by a TLR9 agonist. When we inhibited TLR9‐mediated signalling by using a TLR9 antagonist, BMDMs from WT and MAIR‐II–deficient mice produced comparable levels of pro‐inflammatory cytokines. Thus, our data imply that MAIR‐II activates TLR9‐mediated signalling in macrophages, hence amplifying the pro‐inflammatory response and worsening adverse cardiac outcome after MI (Figure S7). However, the direct role of MAIR‐II in TLR9‐mediated signalling in MI remains unclear. Further in vivo studies are necessary to clarify that MAIR‐II is involved in TLR9‐mediated signalling.

In this study, we focused on the role of MAIR‐II in macrophages because previous studies have shown that dysregulated macrophage activation causes poor cardiac prognosis after MI. However, as we previously reported, MAIR‐II transcripts were expressed in multilineage cells, including lymphohaematopoietic progenitors, granulocytes, macrophages, erythroid cells and B cells in bone marrow, and in T, B and NK1.1^+^ cells in the spleen.^11^ Therefore, MAIR‐II in cells other than macrophages may also be involved in excessive inflammation after MI. Further investigation is necessary to elucidate the exact role of MAIR‐II in MI.

## CONFLICT OF INTEREST

The authors declare no conflicts of interest.

## AUTHOR CONTRIBUTION

**Saori Yonebayashi:** Data curation (lead); Investigation (lead); Writing‐original draft (lead). **Kazuko Tajiri:** Conceptualization (lead); Funding acquisition (lead); Investigation (supporting); Methodology (equal); Project administration (lead); Supervision (lead); Writing‐review & editing (lead). **Nobuyuki Murakoshi:** Writing‐review & editing (supporting). **Dongzhu Xu:** Investigation (supporting); Writing‐review & editing (supporting). **Siqi Li:** Investigation (supporting). **Duo Feng:** Investigation (supporting). **Yuta Okabe:** Investigation (supporting). **Zixun Yuan:** Investigation (supporting). **Zonghu Song:** Investigation (supporting). **Kazuhiro Aonuma:** Investigation (supporting). **Akira Shibuya:** Conceptualization (supporting); Supervision (supporting); Writing‐review & editing (supporting). **Kazutaka Aonuma:** Project administration (supporting); Supervision (supporting); Writing‐review & editing (supporting). **Masaki Ieda:** Conceptualization (supporting); Project administration (supporting); Writing‐review & editing (supporting).

## Supporting information

Fig S1‐S7

## Data Availability

The data that support the finding of this study are available from the corresponding author upon reasonable request.
