# Supplementary material for: MAIR‐II deficiency ameliorates cardiac remodelling post‐myocardial infarction by suppressing TLR9‐mediated macrophage activation
Source: J Cell Mol Med. 2020 Nov 2;24(24):14481–90. doi: 10.1111/jcmm.16070 (PMC7753988; doi:10.1111/jcmm.16070)
Supplement: Supplementary file 1 — Fig S1‐S7 [file JCMM-24-14481-s001.pdf]

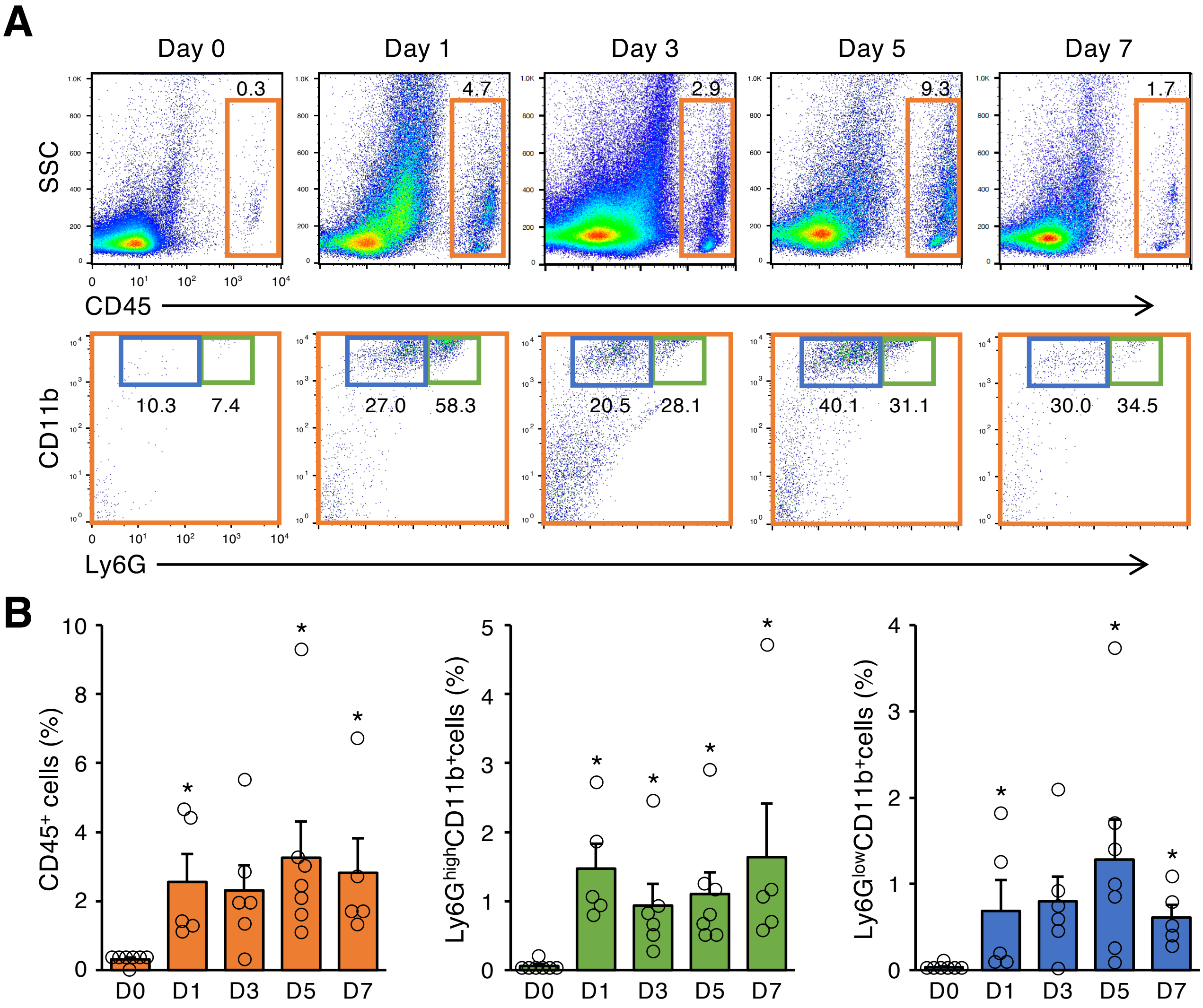

Figure S1. Dynamics of inflammatory cells in infarcted myocardium.
(A) Representative flow cytometric plots showing infiltrating inflammatory cells in infarcted hearts at the indicated time points. 7-AAD negative live cells were gated to determine leukocytes ($CD45^+$ cells, upper panels). Leukocytes were further gated to determine $Ly6G^{high/low}CD11b^+$ myeloid cells (lower panels). (B) Quantification of inflammatory cells from infarcted hearts as a percentage of live cells at the indicated time points (n = 5-7). *P<0.05 vs day 0 by Kruskal-Wallis analysis with a post-hoc Steel test.

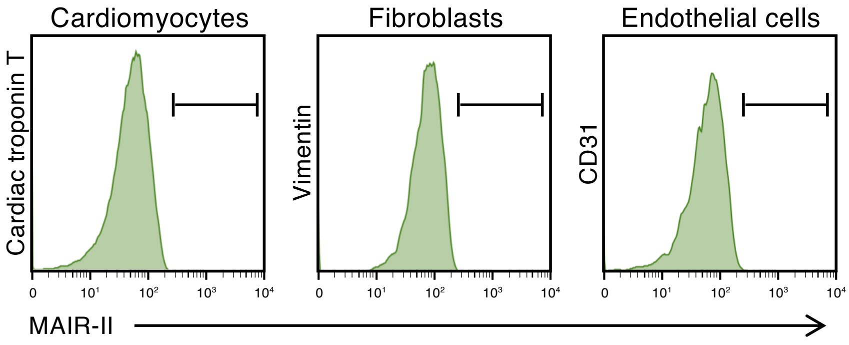

Figure S2. Flow cytometric analysis of MAIR-II expression on cardiomyocytes, fibroblasts, and endothelial cells.

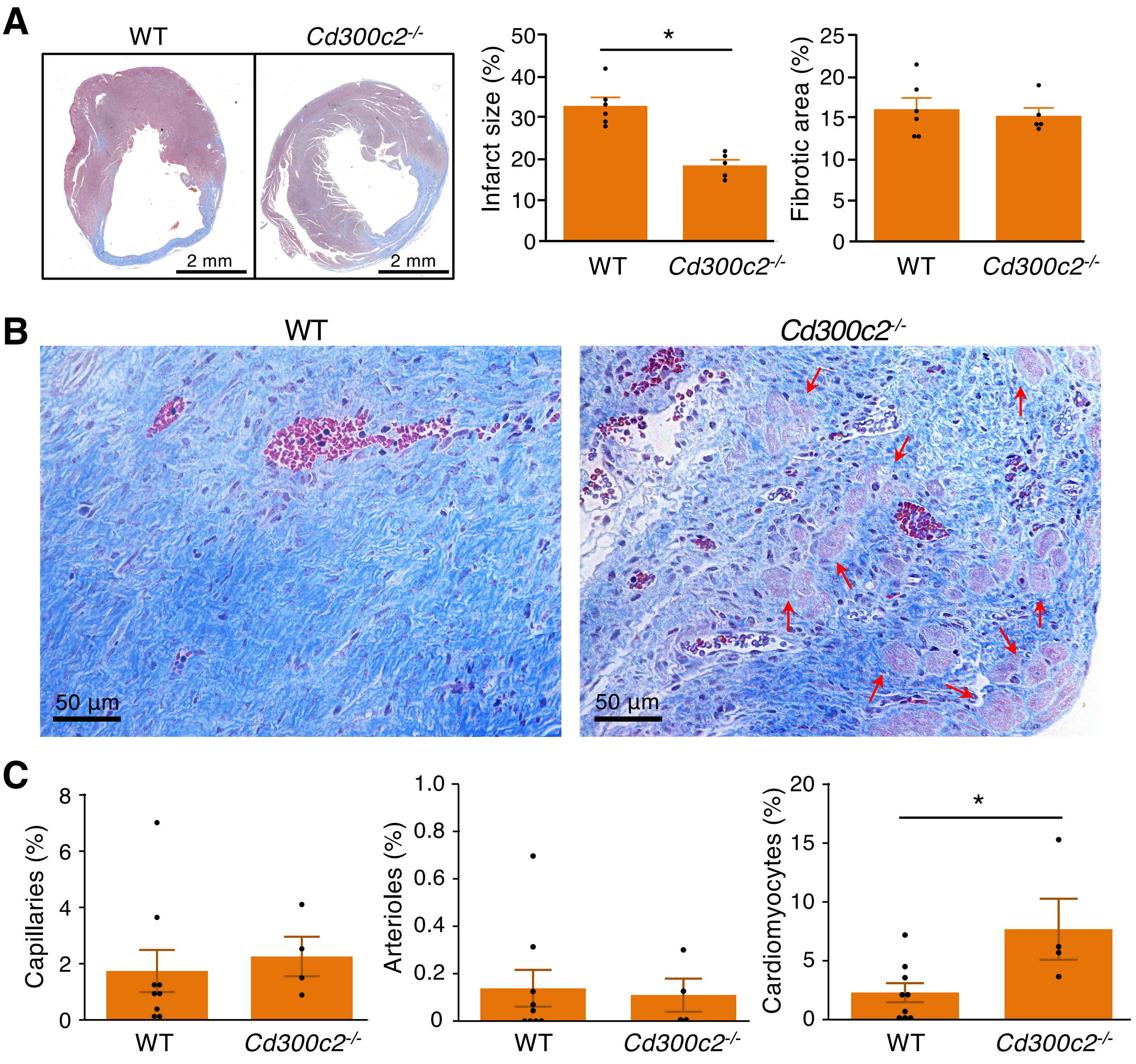

Figure S3. Histological examination of the heart 14 days after MI.
(A) Representative cross sections of heart tissue and the quantification of infarct sizes and fibrotic areas between WT and *Cd300c2*$^{-/-}$. Results are presented as mean ± SEM, n=5 each. (B) Representative cross sections of the infarct area. (C) The quantification of the amount of capillaries, arterioles, and cardiomyocytes in the infarct area 14 days post-MI. Results are presented as mean ± SEM, n = 4-9. *$P < 0.05$ by Mann-Whitney *U* test.

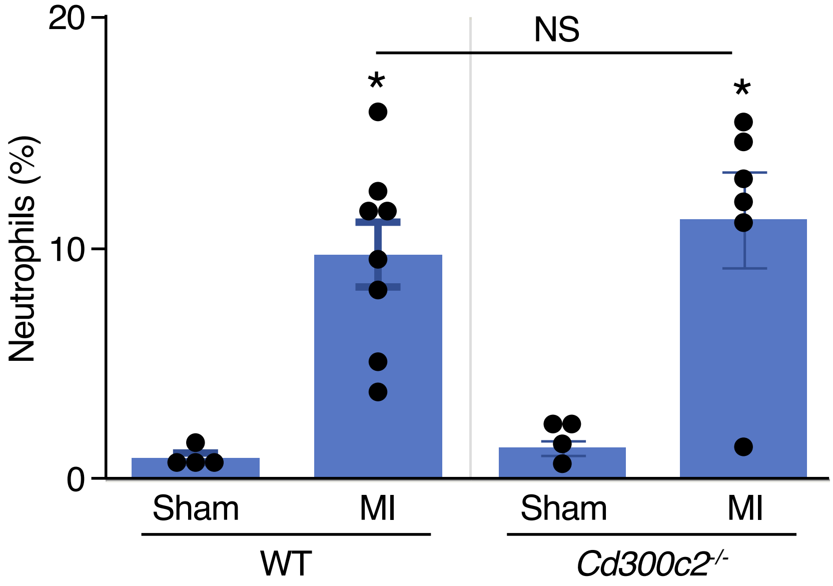

Figure S4. Neutrophil infiltration in the heart.
Quantification of Ly6G^{high}CD11b^{+} neutrophils as a percentage of live cells from WT and *Cd300c2*$^{-/-}$ hearts from sham and day 5 after MI. Results are presented as mean ± SEM, n = 4-8. *$P < 0.05$ vs sham by one-way ANOVA with Tukey's post hoc test.

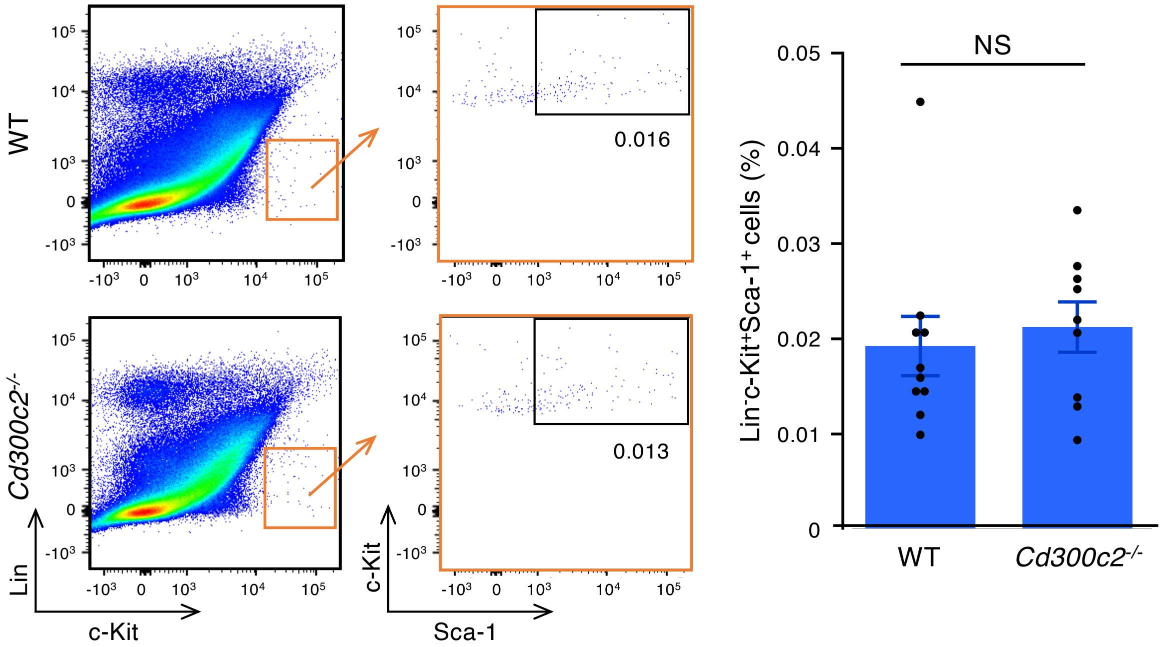

Figure S5. Hematopoietic stem cell infiltration in the heart.
Quantification of $Lin^-c\text{-}kit^+Sca\text{-}1^+$ hematopoietic stem cells as a percentage of live cells in the infarcted hearts from WT and *Cd300c2*$^{-/-}$ mice 8 days after MI. Results are presented as mean ± SEM, n = 9-11.

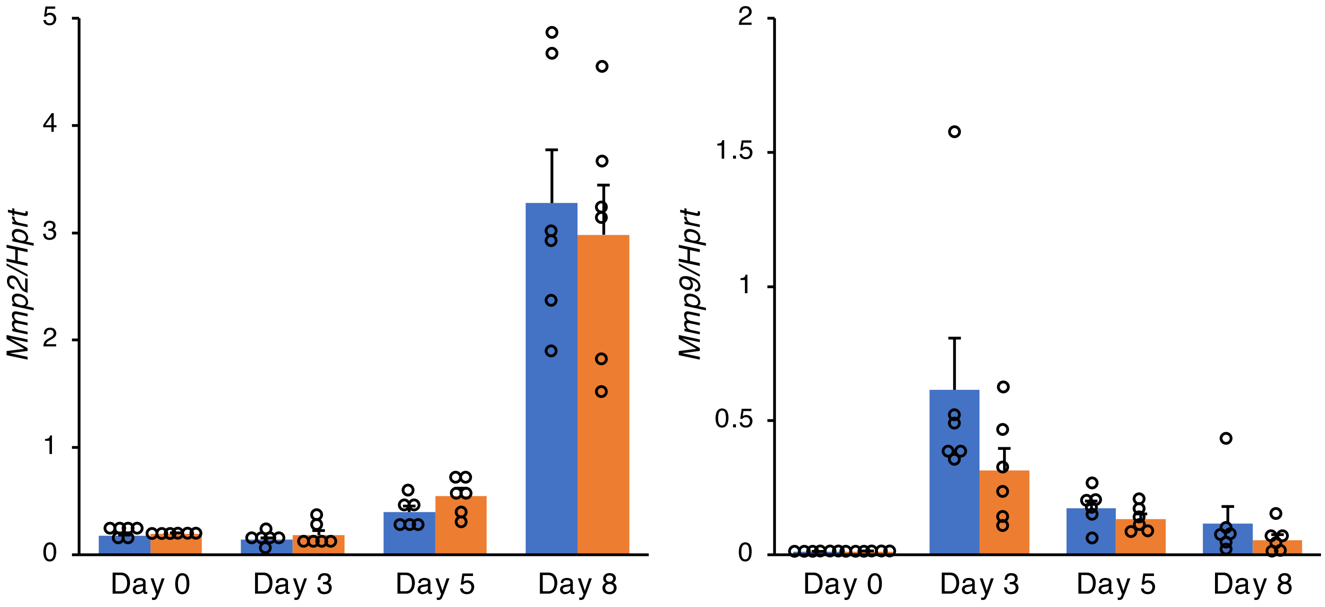

Figure S6. Gene expression of MMPs in infarcted hearts.
mRNA expression of MMPs in hearts from WT and *Cd300c2*$^{-/-}$ hearts. Results are presented as mean ± SEM, n = 6-8.

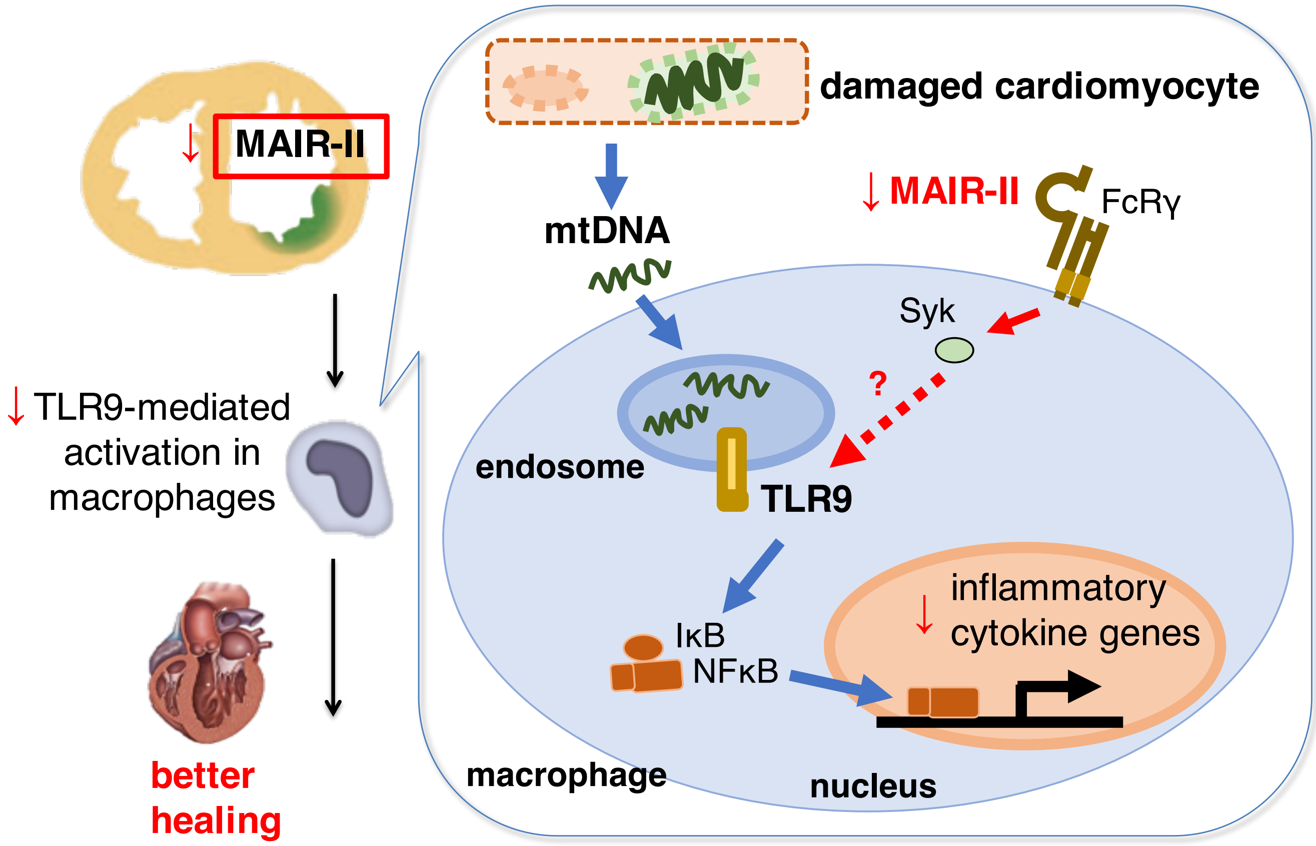

Figure S7. MAIR-II deficiency prevents cardiac dysfunction post-MI.
Damaged cardiomyocytes release mtDNA as a DAMP, which activates endosomal TLR9 in macrophages. Upon TLR9 activation, NF-κB-mediated inflammatory cytokine production is upregulated. However in MAIR-II deficiency, TLR9-mediated activation in macrophages is suppressed and thus a dampened inflammatory response results in better healing post-MI.
